# Strontium enhances proliferation and osteogenic behavior of bone marrow stromal cells of mesenchymal and ectomesenchymal origins in vitro

**DOI:** 10.1002/cre2.221

**Published:** 2019-08-21

**Authors:** Carolina Bizelli‐Silveira, Lisbeth Ann Abildtrup, Rubens Spin‐Neto, Morten Foss, Kjeld Søballe, David Christian Evar Kraft

**Affiliations:** ^1^ Department of Dentistry and Oral Health, Faculty of Health Aarhus University Aarhus Denmark; ^2^ Department of Orthopaedic Surgery Aarhus University Hospital Aarhus Denmark; ^3^ Interdisciplinary Nanoscience Center (iNANO), Department of Physics and Astronomy, Faculty of Science and Technology Aarhus University Aarhus Denmark

**Keywords:** bone marrow stromal cells, embryonic origin, osteogenic, strontium

## Abstract

**Obejective:**

To investigate the effect of increasing Strontium (Sr) concentrations on the growth and osteogenic behavior of human bone marrow stromal cells (BMSCs) from mesenchymal (i.e., fibula) and ectomesenchymal (i.e., mandible) embryonic origins.

**Materials and methods:**

Fibula and mandible BMSCs were cultured in media without (Ctrl) or with Sr in four diverse concentrations: Sr1, 11.3 × 10^−3^ mg/L, human seric physiological level; Sr2, 13 mg/L, human seric level after strontium ranelate treatment; Sr3, 130 mg/L, and Sr4, 360 mg/L. Proliferation rate (1, 3, and 7 days), osteogenic behavior (alkaline phosphatase [ALP] activity, 7 and 14 days; expression of osteogenic genes (ALP, osteopontin, and osteocalcin at 7, 14, and 21 days), and formation of mineralized nodules (14 and 21 days) of the BMSCs were assessed. Data was compared group‐ and period‐wise using analysis of variance tests.

**Results:**

Fibula and mandible BMSCs cultured with Sr4 showed increased proliferation rate, and osteocalcin and osteopontin gene expression together with more evident formation of mineralized nodules, compared all other Sr concentrations. For both cell populations, Sr4 led to lower ALP activity, and ALP gene expression, compared with the other Sr concentrations.

**Conclusion:**

BMSCs from mesenchymal (i.e., fibula) and ectomesenchymal (i.e., mandible) embryonic origins showed increased cellular proliferation and osteogenic behavior when cultured with Sr4, in vitro.

## 1 INTRODUCTION

Bone marrow stromal cells (BMSCs) have been suggested as a suitable cellular source for bone tissue engineering therapies (Dayi & Omezli, 2011). However, these cell populations have a heterogeneous nature, which is evident from the broad range of colony sizes, varying growth rates, and different cell morphologies, when cultured (Bianco, Riminucci, Gronthos, & Robey, 2001). One source for these differences among BMSC populations is the fact they can originate from diverse bones in the body (Bianco et al., 2001; Meunier et al., 2004). Whereas mesenchymal progenitor cells isolated from various tissues share many similar characteristics, they exhibit minor differences in their expression profile and differentiation potential (Rastegar et al., 2010). This implies that they might even come from different embryonic origins (i.e., mesenchymal or ectomesenchymal), and have diverse differentiation capacity, also being diversely regulated by external factors (Bianco et al., 2001; Meunier et al., 2004).

Although the axial and the appendicular skeleton has mesenchymal embryonic origin, the cranio‐facial skeleton has ectomesenchymal origin (Shorr & Carter, 1952). Although the same key regulators of osteoblastic differentiation determine precursor commitment in bone tissue independent of its embryonic origin, several growth factors, receptors, and associated signaling cascades play distinct roles in the cranio‐facial versus the axial and appendicular skeleton (Abzhanov, Rodda, McMahon, & Tabin, 2007). These developmental differences imply the existence of site‐specific properties of progenitor cells in bone marrow (Akintoye et al., 2006). Added evidence that orofacial bone development differs from that of axial and appendicular bone formation is suggested by the existence of skeletal diseases such as cherubism (Ueki et al., 2001) and hyper parathyroid jaw tumor syndrome (Simonds et al., 2002), which affect only bones of ectomesenchymal origin.

Substances able to enhance the proliferation and differentiation of BMSCs into bone‐producing cells (i.e., osteoblasts) are of interest for bone tissue engineering studies (Bianco et al., 2001; Dayi & Omezli, 2011; Krebsbach, Kuznetsov, Bianco, & Robey, 1999). Following a long list of possible agents, strontium (Sr) performs as a reasonable candidate (Andersen et al., 2013; Park, Kim, Jang, & Song, 2013). First suggested as an adjunct for osteoporosis treatment, Sr performs two possible actions in bone tissue, i.e., stimulate bone formation and suppress bone resorption (Burlet & Reginster, 2006). Systemic treatment with Sr is proposed to enhance bone defect regeneration, and implant osseointegration (Eriksen, 1986). The local delivery of Sr from functionalized implants and graft materials is also a possibility addressed in the literature (Andersen et al., 2013; Lips, Courpron, & Meunier, 1978). The pathway determining the anabolic effect of Sr on bone remains poorly understood. *In vitro* studies suggest that Sr can promote osteoblast proliferation and osteoblastic differentiation in populations rich in precursor cells, such as BMSCs (Bianco et al., 2001; Choudhary, Halbout, Alander, Raisz, & Pilbeam, 2007; Friedenstein, Latzinik, Grosheva, & Gorskaya, 1982; Peng et al., 2009), human adipose‐derived stem cells (Aimaiti et al., 2017), and human periodontal ligament cells (PDLCs; Bizelli‐Silveira et al., 2018).

Previous studies in the literature presented the bone anabolic effect of Sr at the cellular level (Bonnelye, Chabadel, Saltel, & Jurdic, 2008; Canalis, Hott, Deloffre, Tsouderos, & Marie, 1996; Lips et al., 1978; Zhu et al., 2007). Sr has been shown to enhance cell replication and functional activities, including bone matrix synthesis and alkaline phosphatase (ALP) activity of mouse and rat preosteoblasts and osteoblasts (Bonnelye et al., 2008; Canalis et al., 1996; Choudhary et al., 2007; Zhu et al., 2007). Several osteogenic genes can be up‐regulated upon Sr treatment, such as Runt‐related transcription factor 2 (RUNX2), bone sialoprotein (BSP), ALP, osteopontin (OPN), and osteocalcin (OCN; Lips et al., 1978). It has been suggested that Sr exerts its anabolic effects on bone formation in a dose‐dependent manner (Bizelli‐Silveira et al., 2018; Brennan et al., 2009; Marie, 2007). Rats BMSC osteoblastic differentiation was enhanced when the culture medium was supplemented with 1 mM (8.7 mg/L) of strontium ranelate (SrRan), compared with 0.1 mM (87 mg/L; Marie, 2007). A recent study using PDLCs showed higher OCN and OPN gene expression, together with more evident formation of mineralized nodules, when the medium was supplemented with 360 mg/L of Sr, compared with lower Sr concentrations (Bizelli‐Silveira et al., 2018). On the other hand, a study using rat primary osteoblasts showed that Sr‐supplementation exceeding 20 g/L inhibited the formation of hydroxyapatite (Brennan et al., 2009). As for now, no consensus on the ideal Sr‐supplementation concentration has been established in the literature.

Thus, the objective of the present study was to investigate the effect of increasing Sr concentrations on the growth and osteogenic behavior of human BMSCs of mesenchymal (i.e., fibula) and ectomesenchymal (i.e., mandible) embryonic origins.

## 2 MATERIAL AND METHODS

### 2.1 Primary cell culture

Primary human bone cells were isolated from the fibula and from the mandible of two healthy patients. From these patients, primary cell‐explant cultures were established for further investigation (donor fibula: male, 46 years old; donor mandible: male, 46 years old). Specimens were obtained under informed consent according to the guidelines of the Central Denmark Region Committee on Biomedical Research Ethics. Tissue was collected from patients undergoing orthopedic bone grafting surgery, the obtained samples consisted of material that would otherwise be discarded. The samples were delivery to the laboratory without patient referable information other than sex and age, and therefore considered a health research project using anonymous adult cell lines. Danish regulatory requirements, regarding health research project with anonymous adult cell lines is exempted from approval by a regional ethics committee.

The methodology used in the present study follows, in detail, a methodology previously used by our group to establish the optimal concentration of Sr to enhance the osteogenic behavior of PDLCs (Bizelli‐Silveira et al., 2018). After retrieval, the bone samples from the fibula and from the mandible were immediately stored in Minimum Essential Medium (MEM)‐filled sterile tubes (Sigma‐Aldrich, St. Louis, USA). The pieces of bone were cleaned thoroughly with phosphate‐buffered saline (Sigma‐Aldrich, St. Louis, USA) and enzymatically, through digesting for 30 min at 37°C in MEM (Sigma‐Aldrich, St. Louis, USA) containing 3 mg/ml collagenase type I (Medinova, Zürich, Switzerland) and 2.4 units/ml dispase II (Roche Diagnostics, Mannheim, Germany). Single BMSC suspensions were obtained by filtration through 70‐μm cell sieves. The isolated BMSCs were hereafter cultured in MEM supplemented with 10% fetal bovine serum (Sigma‐Aldrich, St. Louis, USA) and antibiotics (25.000 IU/ml penicillin and 25 mg/ml streptomycin; DuraScan Medical Products, Odense, Denmark) at 37°C in an atmosphere of 100% relative humidity and 5% CO_2_. Before reaching confluence, BMSCs were trypsinized (Sigma‐Aldrich, St. Louis, USA), harvested, and transferred to new cell culture flasks, subcultured in a humidified 37°C incubator with 5% CO_2._ The medium was changed twice a week. All experiments were carried out with cells of passage 3.

Cell experiments (cell proliferation analysis, osteogenic behavior assessment, and mineralization analysis) were undertaken with the established BMSC populations, following the evaluation periods selected for each of the experiments.

### 2.2 Composition of cell culture media

Cells were cultured without (Ctrl) or with one of four increasing Sr concentrations, according to a previous study (Bizelli‐Silveira et al., 2018), defining the group they were allocated to Sr1, the physiological level of Sr in the human serum (11.3 × 10^−3^ mg/L); Sr2 (human seric concentration reached after culture with a common Sr systemic supplementation used by osteoporotic women, 13 mg/L); Sr3, 10 times the Sr2 concentration (130 mg/L); and Sr4, approximately 30 times the Sr2 concentration (360 mg/L). Sr was added as strontium chloride hexahydrate (SrCl_2_·6H_2_O, Sigma‐Aldrich, St. Louis, USA).

### 2.3 Cell proliferation

BMSCs from the mandible and from the fibula were seeded separately at a density of 15 × 10^3^ cells/cm^2^ in 24‐well plates, divided according to the groups (Ctrl, Sr1‐Sr4). The cells were harvested after 1, 3, 7, and 14 days of culture, trypsinized with 0.25% TrypLE™ Express reagent, no‐phenol red (Gibco, Paisley, UK), and counted using NucleoCounter™ (ChemoMetec, Allerod, Denmark). The cell count was made using 100 μl of culture sample, and according to NucleoCassete™ manufacturer's instructions.

### 2.4 ALP activity assay

BMSCs were seeded at 15 × 10^3^ cells/cm^2^ in 96‐well plates. At indicated time points (7 and 14 days of culture), cultures were washed with phosphate buffered saline and lysed with alkaline buffer containing 1.5 M 2‐amino‐2‐methyl‐1‐propanol (100 μl/well; Sigma‐Aldrich, St. Louis, USA), pH 10.3, for 10 min at 37°C. Then, 100 μl pNPP substrate solution (1 mg/ml pNPP substrate in 0.1 M Glycin buffer; Sigma‐Aldrich, St. Louis, USA) was added to the cell lysate for 2.5 min at 37°C, followed by addition of 100 μl 2 M NaOH for 10 min to stop the enzymatic ALP conversion of pNPP into pNP. Spectrophotometrical quantification of pNP was performed on an EL800 absorbance microplate reader (BioTek, Winooski, USA) at a wavelength of 405 nm. For a standard curve, 1‐mM stock pNP was diluted from 0.05 to 0.4 mM pNP (Sigma‐Aldrich, St. Louis, USA) and used to calculate cell‐specific ALP activity, which was expressed as mM pNP/minute/cell. The pNPP substrate and the 2 M NaOH stop solution were added at the same time as to the samples, providing the standard curve. Data were calculated for Sr and Ctrl groups after 7 and 14 days of culture.

### 2.5 Gene expression–RNA isolation and real‐time polymerase chain reaction analysis

BMSCs were studied for their osteogenic gene expression after culture with Ctrl and Sr1–4. Real‐time polymerase chain reaction (RT‐PCR) was performed to evaluate the mRNA levels of osteogenesis‐related genes ALP, OCN, and OPN. For RT‐PCR, BMSCs in Sr and Ctrl groups were seeded at 15 × 10^3^ cells/cm^2^ in 6‐well plates, in triplicate for each source of BMSCs. After 7, 14, and 21 days of culture, BMSCs were harvested and total RNA was extracted and purified using Macherey‐Nagel total RNA kit (Macherey‐Nagel, Düren, Germany) according to the manufacturer's instructions. RNA concentration and purity were spectrophotometrically determined using an Eppendorf BioPhotometer (Eppendorf, Hamburg, Germany) according to the manufacturer's instructions. The RNA samples were treated with recombinant DNase I (Macherey‐Nagel, Düren, Germany) and converted into cDNA using cDNA synthesis kit (cat no. 600559, Agilent Technologies, Santa Clara, USA). RT‐PCR was performed on a Stratagene Mx3000P system (Stratagene, San Diego, USA) using TaqMan universal PCR master mix (Applied Biosystems, Waltham, USA) and TaqMan gene expression assays (Applied Biosystems, Waltham, USA) with the following primers: secreted phosphoprotein 1 (*SPP1*) Hs00959010_m1 (OPN), bone gamma‐carboxyglutamate (gla) protein (*BGLAP*) Hs01587814_g1 (OCN), and ALP‐liver/bone/kidney (*ALPL*) Hs00758162_m1 (ALP; Applied Biosystems, Waltham, USA). Standard enzyme and cycling conditions for the Stratagene Mx3000P system were used. Template cDNA corresponding to 1.176 × 10^−2^ μg of RNA was added to each PCR reaction, and each biological sample was run in technical duplicates for each gene. Data analysis was performed using Stratagene Mx3000P RT‐PCR system sequence detection software version 1.3 (Stratagene, San Diego, USA). Expression levels of the gene of interest were normalized to the “BestKeeper” index (Pfaffl, Tichopad, Prgomet, & Neuvians, 2004), determined by the geometric mean of threshold cycles (Ct) from ribosomal protein L13a, glucuronidase, and beta and beta‐2‐microglobulin.

### 2.6 Mineralization using alizarin red staining

BMSCs from the different sources were seeded at 15 × 10^3^ cells/cm^2^ in 24‐well plates. Alizarin red staining (ARS) of BMSC cultures was carried out after 14 and 21 days of culture. BMSCs were washed with phosphate buffered saline and fixed with 70% ethanol for at least 1 hr at −20°C. The fixed BMSCs were washed with double distilled water (ddH_2_O) and stained with 0.2% Alizarin Red (Sigma‐Aldrich, St. Louis, USA) for 15 min, with rotation and at room temperature. Then the Alizarin Red solution was carefully aspirated and the cell monolayer was washed 5 times with ddH_2_O to remove nonspecific staining. The cells were then air‐dried. Images of the remaining red‐orange spots, formed by the chromogenic complex between o‐cresolphthaleon and calcium ions, were captured at 10x magnification using a light microscope (Olympus IX73, Tokyo, Japan) and a digital camera (Olympus, Tokyo, Japan). A previous study from our group (Bizelli‐Silveira et al., 2018) tested a control containing Sr4, but with no cells, to allow the visualization of false‐positive results due to possible interaction between Sr in high concentrations and the ARS. Possible cell‐independent binding of Sr to the culture well surfaces has been found not to be detectable by ARS. And therefore this test was not performed in the present study.

### 2.7 Statistical analysis

All experiments were performed in triplicate (i.e., in all experiments, three samples per group/period were tested). The data were described as means and standard deviations. The statistical protocol was designed according to cell culture studies using similar methodology. Normality of the data was tested and confirmed with the Kolgomorov–Smirnov test, and comparisons (group‐wise and period‐wise) were made using Tukey post hoc test followed a parametric one‐way analysis of variance. The minimum statistical significance was set at *p* ≤ .05. GraphPad Prism 7.02 for Windows (GraphPad Software Inc., La Jolla, USA) was used for the statistical evaluation.

## 3 RESULTS

### 3.1 Cell proliferation

**Figure 1:**
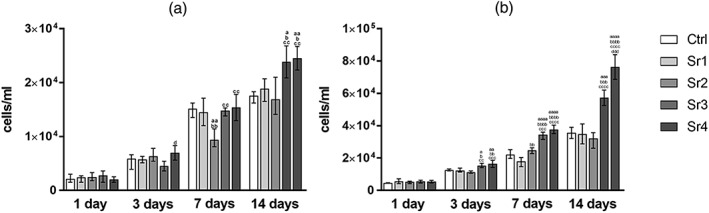
(a) Mandible and (b) fibula bone marrow stromal cell proliferation (mean ± SD) for the control and the Sr1–4 concentrations at the four evaluation periods (n = 3 samples per group/period). The letters on top of each bar indicate statistical difference among the groups within the same period of evaluation. a – Difference from Ctrl, p ≤ .05; aa – difference from Ctrl, p ≤ .01; aaa – difference from Ctrl, p ≤ .001; aaaa – difference from Ctrl, p ≤ .0001; b – difference from Sr1, p ≤ .05; bb ‐ difference from Sr1, p ≤ .01; bbb – difference from Sr1, p ≤ .001; bbbb – difference from Sr1, p ≤ .0001; cc ‐ difference from Sr2, p ≤ .01; ccc – difference from Sr2, p ≤ .001; cccc – difference from Sr2, p ≤ .0001; d – difference from Sr3, p ≤ .05; ddd – difference from Sr3, p ≤ .001; Tukey post‐hoc test followed analysis of variance

For the ectomesenchymal cell population (mandible cells), as shown in Figure 1a, for all Sr concentrations tested, as well as the control, cell counts increased significantly according to the assessment period (i.e., from 1 to 3, from 3 to 7, and then from 7 to 14 days of culture, *p* ≤ .05). Considering the differences among the tested concentrations within the same assessment period, Sr3 and Sr4 had significantly higher number of cells compared with the lower Sr concentrations from 7 days, as showed in Figure 1a.

For the mesenchymal cell population (fibula cells), as shown in Figure 1b, for all Sr concentrations tested, as well as the control, cell counts increased significantly according to the assessment period (i.e., from 1 to 3, from 3 to 7, and then from 7 to 14 days of culture, *p* ≤ .05). Considering the differences among the groups within the same period of evaluation, Sr3 and Sr4 showed significantly larger number of cells compared with the lower Sr concentrations and the control group from 3 days (*p* ≤ .05 for Sr3 and *p* ≤ .01 for Sr4). Subsequently, on day 14, the proliferation was significantly more detectable in the cultures with higher Sr concentration (Sr3 and Sr4) when compared with the other groups (*p* ≤ .001).

### 3.2 ALP activity

**Figure 2:**
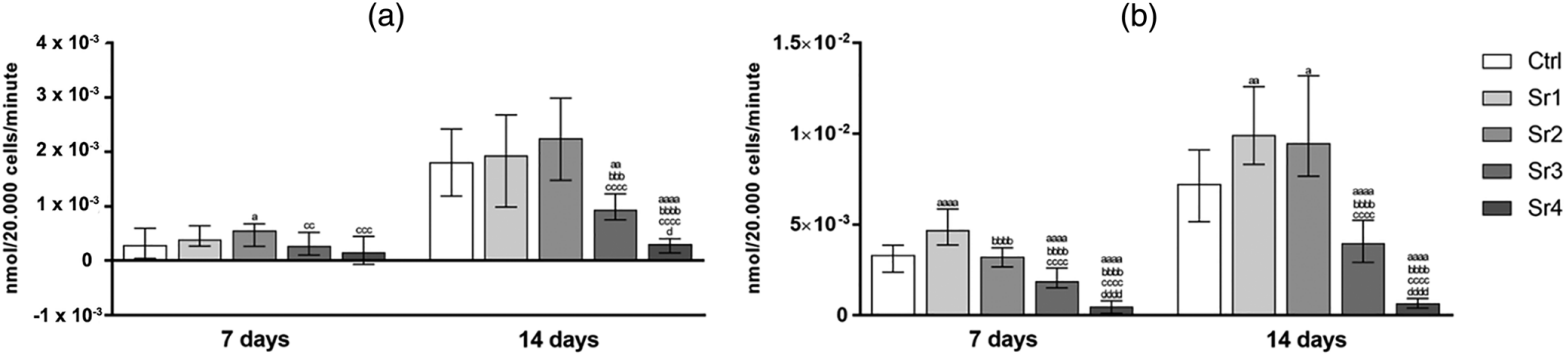
Alkaline phosphatase activity of (a) the mandible and (b) the fibula fibula bone marrow stromal cells (mean ± SD) for the control and the Sr1–4 concentrations at the two evaluation periods (n = 3 samples per group/period). The letters on top of each bar indicate statistical difference among the groups within the same period of evaluation. a – difference from Ctrl, p ≤ .05; aa – difference from Ctrl, p ≤ .01; aaaa – difference from Ctrl, p ≤ .0001; bbb – difference from Sr1, p ≤ .001; bbbb – difference from Sr1, p ≤ .0001; cc ‐ difference from Sr2, p ≤ .01; ccc – difference from Sr2, p ≤ .001; cccc – difference from Sr2, p ≤ .0001; d – difference from Sr3, p ≤ .05; dddd – difference from Sr3, p ≤ .0001; Tukey post hoc test followed analysis of variance

Considering the mandible cells, Sr significantly stimulated the initial rate of p‐NPP hydrolysis over time (from 7 to 14 days) in the mandible BMSC culture (*p* ≤ .0001), except Sr4, which did not differ statistically over time. Sr4 and Sr3 showed significantly lower ALP activity at 14 days when compared with Ctrl and other Sr groups (i.e., Sr1, Sr2, and Ctrl, *p* ≤ 0.01 for Sr3 and *p* ≤ 0.0001 for Sr4) in the same period, as it can be seen in Figure 2a.

Regarding the fibula cells, for all groups except Sr4, Sr significantly stimulated the initial rate of p‐NPP hydrolysis over time (from 7 to 14 days) in the fibula BMSC culture (*p* ≤ .0001). Sr4 and Sr3 showed significantly lower ALP activity, particularly at 14 days, when compared with Ctrl and other Sr groups (i.e., Sr1, Sr2, and Ctrl, *p* ≤ .0001) in the same period, as it can be seen in Figure 2b.

### 3.3 Gene expression

**Figure 3:**
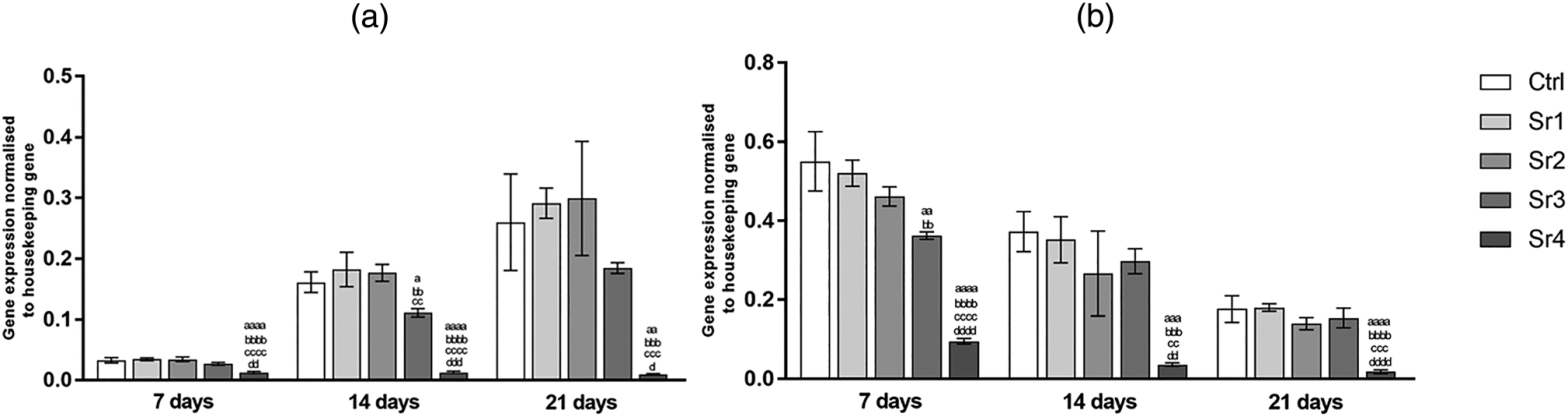
Alkaline phosphatase gene expression of (a) the mandible and (b) the fibula bone marrow stromal cells (mean ± SD), assessed by real‐time polymerase chain reaction (n = 3 samples per group/period). The letters on top of each bar indicate statistical difference among the groups within the same period of evaluation. a – difference from Ctrl, p ≤ .05; aa – difference from Ctrl, p ≤ .01; aaa – difference from Ctrl, p ≤ .001; aaaa – difference from Ctrl, p ≤ .0001; bb – difference from Sr1, p ≤ .01; bbb – difference from Sr1, p ≤ .001; bbbb – difference from Sr1, p ≤ .0001; cc – difference from Sr2, p ≤ .01; ccc – difference from Sr2, p ≤ .001; cccc – difference from Sr2, p ≤ .0001; d – difference from Sr3, p ≤ .05; dd – difference from Sr3, p ≤ .01; ddd – difference from Sr3, p ≤ .001; dddd – difference from Sr3, p ≤ .0001; Tukey post hoc test followed analysis of variance

For the mandible cells, expression of ALP, as seen in Figure 3a, the gene expression for Sr4 group had a trend similar time‐dependently, whereas for the other groups tested, as well as the control, ALP expression decreased significantly according to the assessment period (i.e., from 7 to 14 and then from 14 to 21 days of culture, *p* ≤ .05). Considering the differences among the groups within the same period of evaluation, Sr4 lead to values that were several times lower than those were found for the other Sr concentrations and the control group (at least *p* ≤ .01, *p* ≤ .001, and *p* ≤ .05, respectively).

Considering the fibula cells, the gene expression of ALP, as seen in Figure 3b, for all tested groups decreased time‐dependently (i.e., from 7 to 14 and then from 14 to 21 days of culture, at least *p* ≤ .05). Besides that, Sr4 demonstrated lower ALP gene expression in each tested period when compared with other groups, as shown in the Figure 3b.

**Figure 4:**
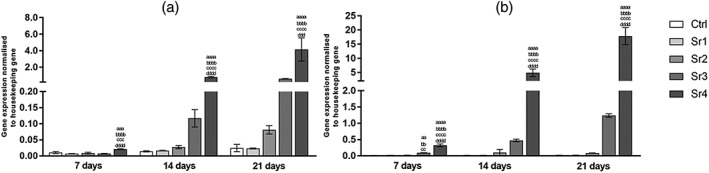
Osteopontin gene expression of (a) the mandible and (b) the fibula bone marrow stromal cells (mean ± SD), assessed by real‐time polymerase chain reaction (n = 3 samples per group/period). The letters on top of each bar indicate statistical difference among the groups within the same period of evaluation. aa – difference from Ctrl, p ≤ .01; aaa – difference from Ctrl, p ≤ .001; aaaa – difference from Ctrl, p ≤ .0001; bb – difference from Sr1, p ≤ .01; bbbb – difference from Sr1, p ≤ .0001; cc – difference from Sr2, p ≤ .01; ccc – difference from Sr2, p ≤ .001; cccc – difference from Sr2, p ≤ .0001; ddd – difference from Sr3, p ≤ .001; dddd – difference from Sr3, p ≤ .0001; Tukey post hoc test followed analysis of variance

**Figure 5:**
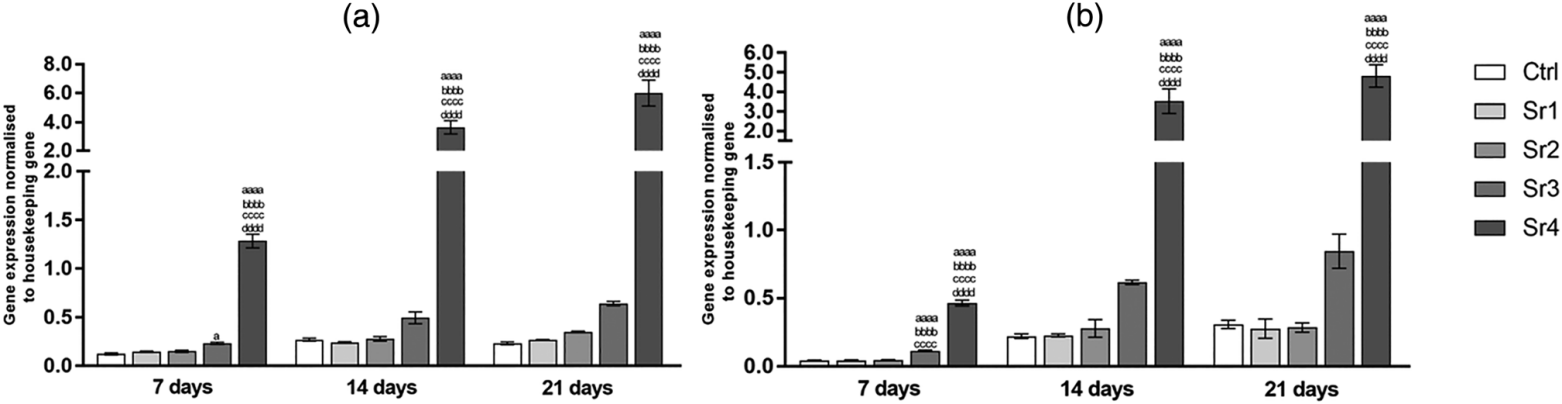
Osteocalcin gene expression of (a) the mandible and (b) the fibula bone marrow stromal cells (mean ± SD), assessed by real‐time polymerase chain reaction (n = 3 samples per group/period). The letters on top of each bar indicate statistical difference among the groups within the same period of evaluation. a – difference from Ctrl, p ≤ .05; aaaa – difference from Ctrl, p ≤ .0001; bbbb – difference from Sr1, p ≤ .0001; cccc – difference from Sr2, p ≤ .0001; dddd – difference from Sr3, p ≤ .0001; Tukey post hoc test followed analysis of variance

For the mandible cells, expression of the late osteoblastic differentiation markers OPN and OCN was both highly up‐regulated in Sr4 (Figures 4a and 5a, respectively) from 7 days, compared with the other groups and control (at least *p* ≤ .001 for OPN and *p* ≤ .0001 for OCN) at the same assessed period. Although control group had a similar trend over time, Sr tested groups showed OPN expression increased from 7 to 21 days, the Sr4 group showed a greater development, with higher concentration being found at 14 and 21 days when compared with day 7 (Figure 4a). OCN expression showed a peak at 7 day for Sr4, attaining higher values after that (7 days to 14 days, *p* ≤ .01 and 7 days to 21 days, *p* ≤ .001) and this is can be observed in Figure 5a.

For the fibula cells, the levels of mature osteoblast gene expression are shown in Figures 4b and 5b. For Sr4, OPN and OCN expressions increased with incubation time (at least *p* ≤ .001 for OPN and *p* ≤ .05 for OCN). At the same assessed period, OPN and OCN revealed the effect being pronounced more intensely in Sr4 from 7 days (*p* ≤ .0001 for OPN and OCN) and these results could be observed in Figures 4b and 5b.

### 3.4 Mineralization

**Figure 6:**
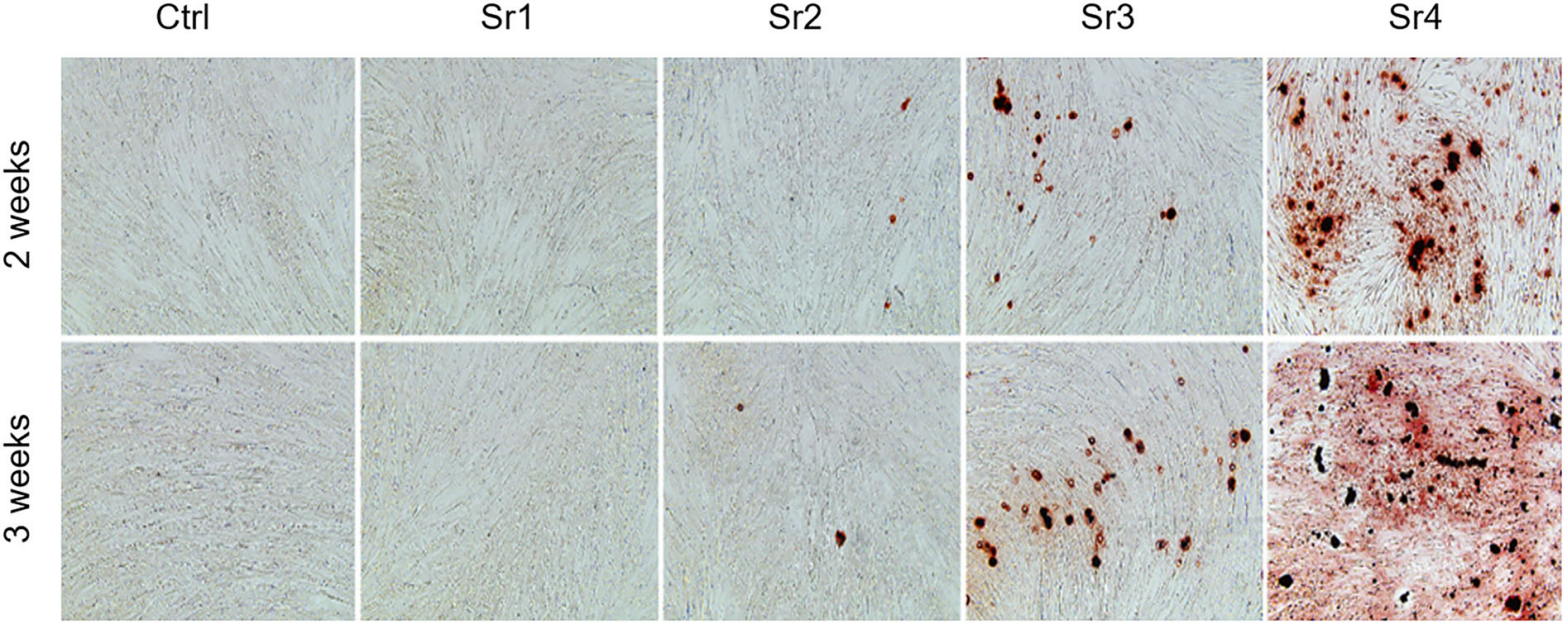
Representative images of the alizarin red staining from the mandible for Ca deposits (bone nodules) for the Sr1–4 concentrations at the two evaluation periods (n = 3 samples per group/period). Magnification rate, 10x

**Figure 7:**
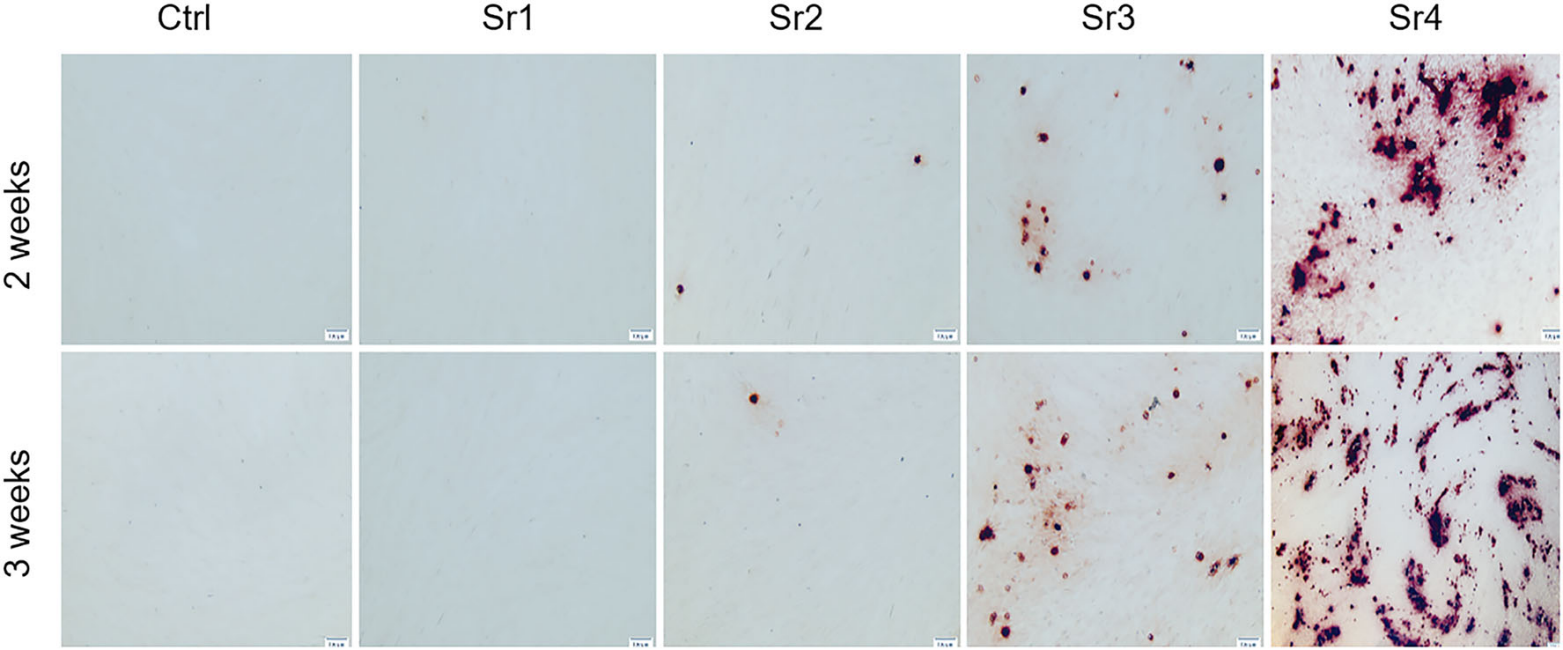
Representative images of the alizarin red staining from the fibula for Ca deposits (bone nodules) for the Sr1–4 concentrations at the two evaluation periods (n = 3 samples per group/period). Magnification rate, 10x

Mineralization, as assessed by subjective alizarin‐red‐positive nodules formed in the mandible and the fibula after 14 and 21 days of culture, was markedly more evident and in larger quantity for the Sr4 group for the mandible and the fibula cell populations, when compared with the other groups, although some positive nodules were also seen for Sr3 (Figures 6 and 7).

## 4 DISCUSSION

Stem cells are undifferentiated cells capable of both self‐renewal and differentiation into diverse cell lineages (Gronthos, Akintoye, Wang, & Shi, 2006). Multipotent stem cells originating from the bone marrow stroma, or BMSCs, are a particularly attractive source for osteogenic precursors for bone tissue engineering (Krebsbach et al., 1999), because they can be easily harvested and expanded in vitro and induced to differentiate into bone‐forming cells.

With an increasing number of older people, the aging “baby boomer” generation, and the increasing life expectancy in developed countries, there is an increased clinical need for effective bone‐regeneration treatment options to repair skeletal defects caused by trauma and disease (Guise & Mundy, 1998; Mackenzie & Flake, 2001). Current bone‐regenerative techniques, based on the use of autologous bone grafting, allografts, and alloplastic bone‐substitute materials, have limitations that hinder their use in a wider range of clinical conditions (Gronthos et al., 2006). Besides that, the development of appropriate delivery approaches for the various growth factors and cells involved in the process (Murphy & Mooney, 1999), is necessary for achieving future viable therapeutic alternatives. The literature (Luu et al., 2009; Mukherjee et al., 2008) indicates that modulation of in vivo lineage differentiation of BMSCs is a feasible approach to build bone, which may serve as a new strategy in those cases in which bone formation is impaired, for example, when the patient suffers from osteoporosis (Meunier et al., 2004).

There are multiple approaches to modulate BMSC differentiation (Aghaloo et al., 2010), including the use of Sr (Peng et al., 2009). The influence of Sr on bone metabolism has been researched since 1950s (Shorr & Carter, 1952). Although previous clinical studies have shown positive effects of Sr supplements on bone formation, the effect of Sr on human BMSCs and osteoblast‐derived human BMSCs has not been elaborated yet.

In the present study, the methodology and the organization and presentation of the results follows, in detail, a previous study from our group that focused on the optimal concentration of Sr to enhance the osteogenic behavior of PDLCs (Bizelli‐Silveira et al., 2018). Here, we used a similar approach on a totally diverse cell population and investigated the effect of increasing Sr concentrations modulating the behavior of human BMSCs. A group of researchers (Sila‐Asna, Bunyaratvej, Maeda, Kitaguchi, & Bunyaratavej, 2007) showed that Sr not only enhances osteogenic differentiation but also shows strong evidence of bone structure stabilization by expressing genes related to bone formation at early day of differentiation, as well as the use of an appropriate concentration for osteogenic induction. Studies (Brennan et al., 2009; Marie, 2007) showed that Sr, administered in the form of SrRan, promotes osteogenesis of mature osteocytes and undifferentiated mesenchymal stem cells in a dose‐dependent manner at concentrations from 10 to 100.000 μM. These Sr concentrations, though, were selected considering the treatment of bone pathologies such as osteoporosis (suggested dose of 2 mg of SrRan per day; Meunier et al., 2009; Reginster et al., 2005). In opposition to that, the Sr concentration (Sr4) used in the present study was selected to be comparable to that reported as possible in an actual in vivo system regarding local delivery of Sr (Andersen et al., 2013) from implant surfaces, and as mentioned, a previous study from our group showed similar effects regarding bone formation for another cell population (PDL cells; Bizelli‐Silveira et al., 2018). In that study, ratifying the osteogenic action of Sr, undifferentiated cells from the periodontal ligament showed higher OCN and OPN gene expression, together with more evident formation of mineralized nodules when the medium was supplemented with high (360 mg/L) Sr concentrations (Bizelli‐Silveira et al., 2018). In the present study, the tested concentrations ranged from the physiological level of Sr in humans to thousands of time this value, but within the limits of what could be actually released from an implant surface, and accumulate in the bone‐to‐implant interface (Andersen et al., 2013).

In the present study, we tested BMSC populations of mesenchymal and ectomesenchymal embryonic origins. It is plausible that observed skeletal site‐specific differences of human BMSCs are related to their different embryological origins and adaptation to functional demands at each skeletal site. Other extrinsic and intrinsic factors that may lead to observed differences include local vascular supply, the cellular composition of the marrow microenvironment, hormonal effects, and muscular attachments that directly accentuate biochemical strains of mechanical load (Eriksen, 1986; Lips et al., 1978). In summary, in the present study, one of the methodologies to evaluate these difference was to quantify the expression of some osteogenesis‐related genes (ALP, OCN, and OPN), following treatment with increasing Sr concentrations.

Our results show the higher Sr concentration tested (Sr4), enhanced growth, and osteogenic behavior (i.e., gene expression and mineralization) of BMSCs from mesenchymal and ectomesenchymal embryonic origins, considering all used assessment methodologies. The capacity of the BMSCs to differentiate into bone‐forming cells was assessed indirectly, by checking the expression of genes related to the osteoblastic phenotype. Some markers correlated with the osteoblastic phenotype are, for example, high ALP level, expression of collage type I and noncollagenous proteins (OCN, OPN, etc.), and cell‐mediated formation of a calcified extracellular matrix (Declercq, Verbeeck, De Ridder, Schacht, & Cornelissen, 2005; Olivares, Rodil, & Arzate, 2007). ALP, as a key marker of osteoblast differentiation, has its expression and activity levels lowered when the osteoblasts become mature (Zhou, Li, Lu, Zhang, & Han, 2013). This takes place because ALP is responsible for the hydrolysis of phosphate esters, which increases the availability of phosphates that react with Ca^2+^ and ultimately trigger the mineralization of collagenous matrices within the bone tissue (Beertsen & van den Bos, 1992). In the present study, during the process of osteogenic differentiation, exposing the cells to a high concentration of Sr (Sr4) downregulated the expression of ALP, but significantly increased the expression of OCN and OPN, and enhanced the formation of calcified nodules for both cell populations studied.

The ALP activity and gene expression results support the idea of an enhanced trail for BMSCs to become mature osteogenic cells. Other studies with multipotent cells suggest that when cells proliferate in an augmented manner (as seen for Sr4), it downregulates ALP gene expression (Choi, Noh, Park, Lee, & Suh, 2011; Lian & Stein, 1995; Stein & Lian, 1993). In other words, during the immediate postproliferative period (from 12 to 18 days), the extracellular matrix undergoes a series of modifications in composition and organization that renders it competent for mineralization. In addition to that, in heavily mineralized cultures, cellular levels of ALP decline (Lian & Stein, 1995).

OPN is expressed both during the period of active proliferation (at 25% of maximal levels), decreases postproliferative, and exhibits induction at the onset of mineralization, achieving peak levels of expression during mineralization (Li et al., 2017; Lian & Stein, 1995). In contrast to OPN, OCN is expressed only after cell culture has become confluent, with the onset of nodule formation, being a late‐stage marker of osteoblast differentiation (Li et al., 2017). The enhanced OPN and OCN gene expression level as well as the deposition of extra cellular calcium demonstrated that Sr, in a proper concentration, could significantly accelerate the differentiation and maturation of the BMSCs, indicating the potential for bone‐tissue healing process in the bone environment.

Besides the assessment of osteogenic marker levels based on gene expression, the determination of in vitro mineralization is also crucial in evaluating terminal osteogenic differentiation of BMSCs. Because Sr is known to exert dose‐dependent increase in mineralization (Bizelli‐Silveira et al., 2018; Brennan et al., 2009; Marie, 2007), it is not surprising to observe robust mineralization of extracellular matrix in the highest concentrations of Sr, as detected by ARS.

## 5 CONCLUSION

From this experimental study, it can be concluded that Sr, in the proper concentration (Sr4), promotes the osteogenic differentiation of BMSCs from mesenchymal (i.e., fibula) and ectomesenchymal (i.e., mandible) embryonic origins, by increased cellular proliferation and expression of multiple genes regulating different osteogenic stages and matrix maturation.

## CONFLICT OF INTEREST

There are no conflicts of interest.

## AUTHOR CONTRIBUTIONS

Carolina Bizelli‐Silveira: corresponding author, data analysis/interpretation, drafting article, statistics, data collection, authored manuscript. Lisbeth Ann Abildtrup: data analysis/interpretation. Rubens Spin‐Neto, Morten Foss, and Kjeld Søballe: study design, statistics, data analysis/interpretation, manuscript drafting. David Christian Evar Kraft: study design, statistics, data analysis/interpretation, manuscript drafting and final revision.
